# Histotripsy Compared With Microwave Ablation, Radiofrequency Ablation, and Stereotactic Body Radiotherapy for the Treatment of Liver Tumors

**DOI:** 10.7759/cureus.100088

**Published:** 2025-12-25

**Authors:** Mihira Mundhava, Bhavin Vadodariya, Pankaj Jangid, Anuj Shah

**Affiliations:** 1 Medicine, Specialty Surgical Oncology (SSO) Cancer Center, Ahmedabad, IND; 2 Surgical Oncology, Specialty Surgical Oncology (SSO) Cancer Center, Ahmedabad, IND; 3 Surgery, Gujarat Cancer Society (GCS) Medical College, Ahmedabad, IND

**Keywords:** hepatocellular carcinoma, histotripsy, image-guided therapy, liver cancer, microwave ablation, minimally invasive oncology, nonthermal ablation, radiofrequency ablation, stereotactic body radiotherapy, ultrasound therapy

## Abstract

Liver cancer encompasses both primary and secondary malignancies, each carrying unique diagnostic and therapeutic challenges. The goals of conventional liver-directed treatments, including radiofrequency ablation, microwave ablation, and stereotactic body radiotherapy, are local tumor control and improved survival outcomes. However, these treatments are limited by the heat-sink effect, incomplete ablation around vascular structures, and risk of collateral tissue injury. These limitations have prompted the exploration of alternative, non-thermal, and minimally invasive options. Histotripsy is a novel ultrasound-based technique that uses focused mechanical cavitation, rather than heat, to fragment tumor tissue, while preserving surrounding bile ducts and blood vessels. Preclinical and early clinical data, including the THERESA and HOPE4LIVER trials, have demonstrated histotripsy's feasibility, safety, and precision, confirming its ability to generate sharply demarcated ablation zones with minimal complications. Without thermal injury, histotripsy offers a vessel-sparing, non-ionizing, and repeatable treatment alternative, especially for patients with lesions adjacent to critical structures or those not suitable for resection or heat-based ablation. This review summarizes current evidence, principles, and translational insights of histotripsy as a promising non-thermal ablative technology in the treatment of hepatic malignancies and highlights its potential role as a future standard in image-guided liver therapy.

## Introduction and background

Liver cancer is the sixth most common malignancy and the third leading cause of cancer deaths in the world. According to the World Cancer Research Fund in 2022, there were 866,136 new instances of liver cancer around the world and 758,725 fatalities from the disease [1]. The burden of liver cancer is highest in Northern Africa and Asia, with the highest mortality incidence. According to the WHO, it’s more common in men than women [1].

**Figure 1:**
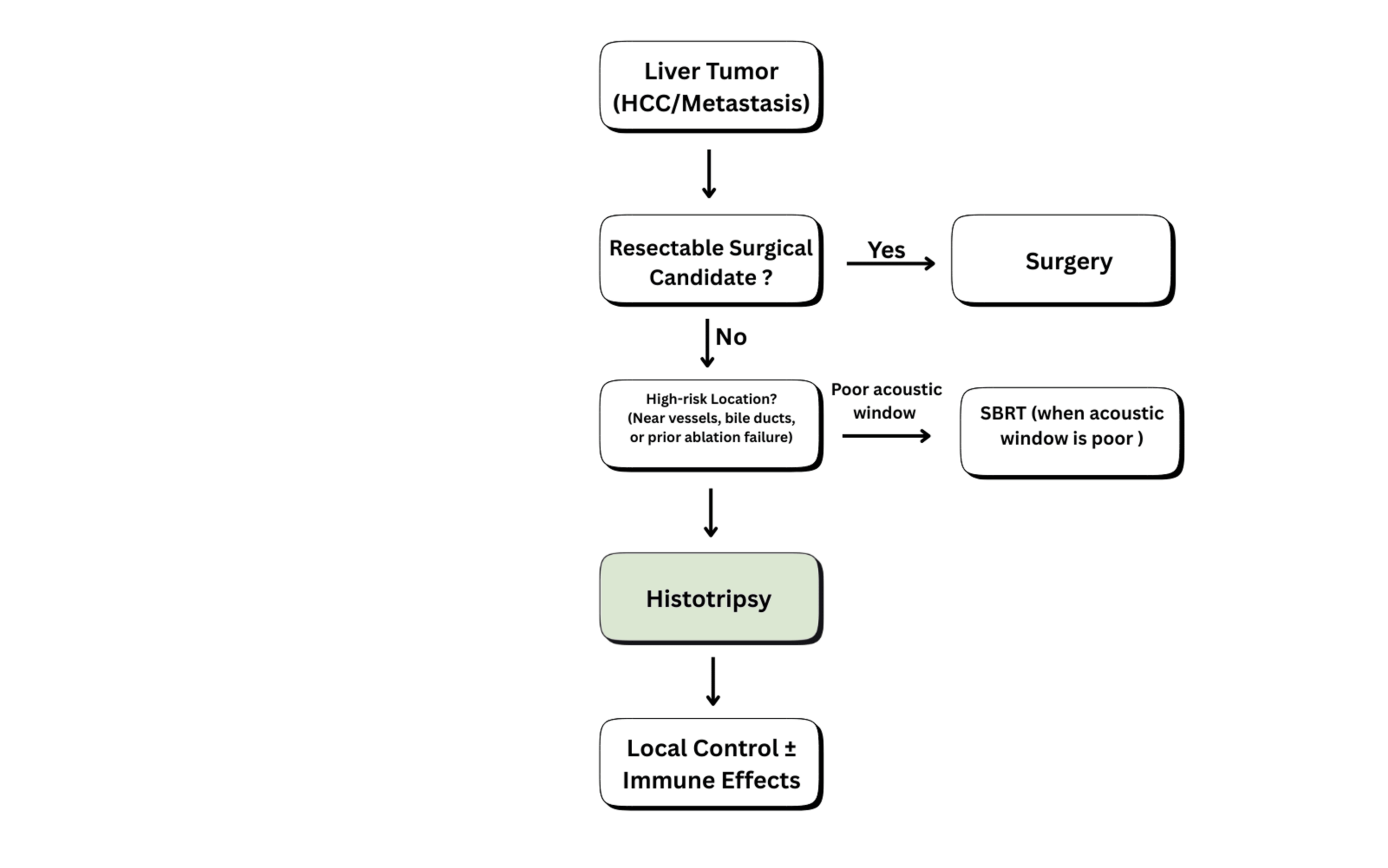
Proposed clinical decision pathway highlighting the role of histotripsy among liver-directed treatment options. HCC: Hepatocellular carcinoma; SBRT: stereotactic body radiotherapy

A key component of the multidisciplinary treatment of primary and secondary hepatic cancers is liver-directed therapy. Surgical resection is still the best way to cure liver cancer, but only 15-25% of patients with liver tumors are good candidates for surgery because they have multiple tumors, hepatic dysfunction, or their tumors are too close to important biliary and vascular structures [2]. Stereotactic body radiotherapy (SBRT) and thermal ablation techniques such as microwave ablation (MWA) and radiofrequency ablation (RFA) are now standard for cases that cannot be surgically operated on or removed. In patients who are not surgical candidates, thermal ablation (RFA/MWA) and SBRT form the backbone of curative-intent local therapy, especially for small, image-visible lesions [3,4]. Current guidance recognizes RFA or MWA as first-line options in very early-stage hepatocellular carcinoma (HCC) (e.g., solitary tumors <2 cm in appropriate candidates), offering meaningful local control with a minimally invasive approach [3,4]. However, in day-to-day practice, their performance is anatomy-dependent: tumors abutting large hepatic vessels are vulnerable to heat-sink-related incomplete necrosis, and lesions near central bile ducts raise the risk of biliary injury, abscess, or bleeding, limitations that persist despite technical refinements [4,5]. SBRT provides a noninvasive alternative when ablation is unsafe or technically limited (e.g., challenging locations, vascular adjacency, or poor ablation window) and modern series report local control rates approaching ~90% in selected HCC cohorts, but treatment is constrained by hepatic reserve and the potential for radiation-related liver toxicity, particularly in cirrhosis [5,6]. Taken together, despite their established clinical roles, these strengths and limitations explain the growing interest in non-thermal, vessel-sparing approaches, setting the stage for histotripsy as a complementary modality rather than a simple replacement. HCC demonstrates marked etiological heterogeneity across geographic regions, with viral hepatitis predominating in Asia and Africa, while metabolic-associated steatotic liver disease (MASLD) is increasingly prevalent in Western populations. These etiological differences influence tumor biology, immune microenvironment, and response to locoregional therapies. Non-thermal ablative approaches such as histotripsy may therefore be particularly relevant in poorly immunogenic (cold) tumors, including MASLD-related HCC, as they preserve native tumor antigen conformation and may enhance immune priming. Histotripsy is a non-thermal focused ultrasound technique that induces mechanical cavitation to fractionate tumor tissue while sparing collagen-rich vascular and biliary structures. Figure 1 summarizes the proposed clinical decision pathway integrating histotripsy into contemporary liver-directed treatment algorithms, particularly for tumors in high-risk locations and for cases following failure of conventional ablation.

Because of these problems, people are more interested in image-guided, non-thermal ablative technologies that can get rid of tumors while leaving healthy tissue around them. The multicenter HOPE4LIVER experiment and the THERESA Phase I research are two early human trials that have proven that histotripsy can be done safely and efficiently on persons with primary or metastatic liver tumors. The technical success rates were over 95%, and there weren't many complications and recovery times were rapid [7]. Preclinical studies indicate that histotripsy may have immunogenic effects, potentially serving as a link between local ablation and systemic immunotherapy. Histotripsy, characterized by its non-thermal mechanism, millimetric precision, and favorable safety profile, possesses the potential to revolutionize the current landscape of liver-directed therapies, particularly for patients with oligometastatic disease, post-systemic downstaging, or small hepatic lesions. This review outlines the principles, existing evidence, comparative benefits, constraints, and prospective clinical applications of histotripsy as an innovative approach for the localized treatment of hepatic malignancies. Histotripsy is a safe, precise, and non-thermal method that can make liver treatment safer and more effective for patients with small or complex lesions.

## Review

Preclinical evidence and translational insights

Experiments on animal liver models have shown that histotripsy works safely and with high precision. Work on pigs and rodents revealed that tissues containing large amounts of collagen tend to resist the cavitation process. When brief, focused ultrasound pulses were applied, they produced clean ablation zones while leaving nearby bile ducts and blood vessels undamaged. In mice with intact immune systems, even treating part of a tumor led to shrinkage of other, untreated tumors elsewhere in the body, suggesting that the effect may involve a broader immune response. In the treatment core, histology always shows a fully acellular homogenate surrounded by patent vasculature and intact.

Histotripsy not only targets and breaks down tumor tissue but also seems to activate the body’s immune system. When tumor cells are mechanically disrupted, they release unaltered tumor antigens along with molecules such as calreticulin, HMGB1, and ATP, collectively called damage-associated molecular patterns (DAMPs). These substances alert and activate dendritic cells, which then produce cytokines like IL-1β and IFN-γ. In mouse models with intact immune systems, partial histotripsy was followed by shrinkage of distant, untreated tumors, pointing to a possible immune-mediated or abscopal effect. It further improves systemic tumor control and CD8⁺ T-cell infiltration when paired with immune checkpoint blockade.

Together, these results show that histotripsy is a promising link between systemic immunotherapy and precise local ablation because it can both "teach" the immune system to identify cancer cells and remove tumors while maintaining essential structures.

Early clinical studies and safety profile

The initial clinical safety of this technology was established by THERESA (Treatment of Hepatic Tumors with Histotripsy), the first-in-human feasibility study. The HistoSonicsprototype system was used to perform percutaneous, ultrasound-guided histotripsy on eight patients with eleven primary or metastatic liver lesions [8]. Without any device-related or Grade 3 adverse events, every procedure was technically successful [8]. Within 36 hours after treatment, an MRI showed clearly defined, non-enhancing cavities that exactly matched the intended ablation zones, while the nearby biliary and vascular structures were still open. Histopathology validated the mechanism of mechanical fractionation in humans by confirming a completely acellular necrotic core with preservation of collagenous septa and vascular scaffolding in the single case that proceeded to liver transplantation [8].

These results were expanded in The Multicenter HOPE4LIVER Trial to 44 patients with 49 liver tumors, including both HCC and metastatic lesions. In this pivotal study, histotripsy achieved technical success in 95% of treated tumors with a low major complication rate in early clinical experience, and no 30-day procedure-related death [9]. The vessel- and duct-sparing characteristic predicted by preclinical models was confirmed by the safe ablation of treated lesions directly next to large hepatic veins or bile ducts. The most common minor events were mild elevations of liver enzymes and temporary right upper-quadrant pain, typically only requiring overnight observation.

These results have been replicated in compassionate-use and registry settings by early institutional series from MD Anderson Cancer Centre, the University of Chicago, and the University of Michigan. This practical experience showed minimal toxicity, quick recovery, and consistent procedural reproducibility across centers. Mild post-procedural pain, localized ecchymosis at the coupling site, and brief increases in AST/ALT were common transient effects that resolved within a week without treatment. Notably, there were no reports of vascular thromboses, thermal injuries, or bile leaks.

Across early reports, histotripsy generally achieved comparable or even higher technical success, with fewer major complications than traditional local treatments. Its non-thermal mechanism circumvents the radiation toxicity that can occasionally be observed with SBRT as well as the heat-sink limitations of RFA and MWA.

Overall, early clinical data support that histotripsy can deliver safe and accurate tumor ablation while maintaining a remarkably low rate of complications. Its potential to supplement-and in some circumstances, replace-current liver-directed modalities is underscored by its repeatability across centers, preservation of vascular and biliary systems, and speedy patient recovery.

Principles

Histotripsy is a non-thermal focused ultrasound technique that uses mechanical cavitation, not heat, to destroy tissue [10]. In contrast to thermal ablation methods such as RFA and MWA, which employ resistive or dielectric heating to induce coagulative necrosis, histotripsy utilizes extremely brief (microsecond-duration), high-amplitude ultrasound pulses to generate a cavitation bubble cloud, or a collection of microscopic vapor bubbles, within the target tissue. The swift growth and subsequent collapse of bubbles generate significant localized shear, shock waves, and microjets when these high-pressure pulses exceed the tissue's inherent cavitation threshold, > 25-30 MPa of negative pressure [10,11]. These mechanical forces break down the treated tissue into a fine, cell-free mixture, yet the nearby parenchyma and connective tissue stay largely intact. These mechanical effects provide sharp ablation margins while sparing surrounding bile ducts and vessels, a major advantage over heat-based modalities. Histotripsy physics has identified two main mechanisms: shock-scattering histotripsy, in which pre-existing microbubbles or rarefaction nuclei act as cavitation seeds that scatter subsequent pulses, maintaining a dense bubble cloud, and intrinsic threshold histotripsy, in which a single or few-cycle pulse crosses the medium's intrinsic threshold to directly initiate cavitation.

Histologically, the treated areas exhibit preserved stromal scaffolding and total acellularity. Granulation tissue and fibrosis gradually replace the homogenate, and there is no longer any indication of thermal artefact. Because the bubble cloud shows up as a short bright spot on B-mode ultrasound, the operator can track treatment progress in real time.

In conclusion, histotripsy is a spatially precise, wholly mechanical method for tissue ablation that reduces collateral damage while eliminating tumors [10]. This important difference from heat-based ablation technologies supports both its possible synergy with immune-mediated tumor control mechanisms and its safety near important vascular and biliary anatomy.

Indication

Primary indications for histotripsy include treating liver tumors that are medically inoperable or unresectable (HCC or metastases) and offering focused local control for oligometastatic disease. Furthermore, a key advantage is that it mitigates the risk of injury or incomplete necrosis from heat-based ablation due to the heat-sink effect in lesions close to major vessels or bile ducts. Histotripsy also plays a vital role in bridge or downstaging therapy before surgical resection or transplantation, and in managing recurrent or residual disease treated after previous SBRT, surgery, or ablation. Additionally, new data points to possible synergy with immune checkpoint inhibitors for both systemic and local control. The major indications for histotripsy in hepatic malignancies are summarized in Table 1 [9].

**Table 1:** Clinical Indications and Advantages of Histotripsy in Liver Tumors

Category	Indication	Key Advantage
Primary tumors	HCC not suitable for resection	Non-thermal, vessel-sparing
Secondary tumors	Oligometastatic liver metastases	Safe near vessels
Critical structures	Near bile ducts / hepatic veins	Reduced thermal injury
Poor surgical candidates	Child-Pugh B/C, comorbidities	Outpatient, safer
Bridge-to-transplant	Control before transplant	Completeness on explant
Experimental	Immunotherapy combinations	Immune stimulation

Contraindication

Due to the non-thermal nature of histotripsy, the absolute and relative contraindications are more limited than those for other image-guided ablative therapies. Hematologic contraindications apply in cases of uncorrectable coagulopathy (platelets < 50,000/\mu\text{L}, INR > 1.5). Systemic contraindications include the inability to administer general anesthesia due to severe cardiopulmonary instability, or the presence of Child-Pugh C or multi-organ failure. Furthermore, focal targeting is not appropriate for diffuse or extensive multifocal disease or tumors larger than 5 cm. Imaging contraindications arise when poor sonographic visualization occurs due to factors like bowel gas, ribs, or an overlying lung preventing an adequate acoustic window, or when coupling is obstructed by massive ascites or an intervening air interface. Pregnancy and implants are special contraindications pending additional safety data. Absolute and relative contraindications for histotripsy are summarized in Table 2 [11].

**Table 2:** Contraindication for Histotripsy in Liver Tumors

Category	Contraindication
Hematologic	Coagulopathy, low platelets
Imaging	Poor sonographic visualization
Tumor spread	Extrahepatic/multifocal disease
Size	Tumour> 5 cm
Location	Adjacent to hollow viscera
Systemic	Child–Pugh C, multi-organ failure
Special	Pregnancy, implants

A comparative summary of histotripsy and conventional ablation methods, MWA, RFA, and SBR, is presented in Table 3. Unlike MWA, RFA, and SBRT, which primarily use heat or radiation and are associated with a major complication rate of 5-10% for thermal methods and 5-8% for SBRT, histotripsy utilizes mechanical cavitation. This results in a low major complication rate in early clinical studies [12-13]. Furthermore, histotripsy demonstrates excellent vessel proximity safety and high immune activation, offering clear advantages over traditional modalities. A comparative summary of histotripsy and conventional ablation methods is presented in Table 3 [8-10,14].

**Table 3:** Comparison of hisototripsy with other treatments

Feature	Histotripsy	MWA	RFA	SBRT
Mechanism	Mechanical cavitation	Heat	Heat	Radiation
Local control (1 yr)	90–95 % (early data)	85–90 %	80–85 %	85–95 %
Vessel proximity safety	Excellent	Poor	Poor	Moderate
Collagenous structure sparing	Yes	No	No	Partial
Immune activation	High	Minimal	Minimal	Variable
Major complications	Major complications: Low (<5%) in early clinical studies	5–10 %	5–10 %	5–8 %
Invasiveness	Non-invasive	Invasive	Invasive	Non-invasive

Procedure of histotripsy 

The ACE aberration correction technique produced a pre-focal bubble cloud [14]. In most histotripsy therapies, this is not an issue, as the target treatment volume generally comprises 1000 or more treatment sites. Consequently, addressing a 2-3 mm margin surrounding a target region, such as a liver tumour, is unlikely to result in significant complications [10]. For histotripsy therapies necessitating great accuracy, such as thrombolysis or brain treatments, a potential option for improving the estimation of pre-focal shockwave production may involve utilizing a B-mode ultrasound imager to assess bubble cloud size [14]. This might subsequently be utilized to implement a more suitable phase calibration to realign the ACE-corrected bubble cloud to the focus [14]. An alternative method for bubble cloud refocusing involves subtracting the phase delays utilized for steering the array prefocally. This approach, by applying solely an axial shift, may excessively generalize the phase correction on the array, thus diminishing focus pressure and bubble cloud size. Nonetheless, it would probably enhance the precision of bubble cloud positioning. These strategies and others will be examined in further investigations [14].

The immediate post-procedure evaluation involves monitoring the patients for 4-6 hours for development of pain, nausea, or hemodynamic instability [8]. Laboratory tests are carried out within 24 hours of the procedure and usually include CBC, Liver Function Tests (AST, ALT, bilirubin, and ALP), and Coagulation Profile-PT/INR [9].

At the stage of intermediate follow-up (3-6 months), attention is turned to detailed tumor assessment, it involves Imaging: MRI or CT every three months for the first six months, monitoring Tumor markers, AFP, CEA, or CA19-9, depending on the type of malignancy [10]. The aim is to confirm the complete absence of residual or recurrent disease, early detection of new lesions, and assessment of immune or inflammatory changes that often result from histotripsy [10].

Long-term follow-up protocols often extend to yearly imaging and biomarker assessments for up to five years, aiming to evaluate the durability of local control and overall survival benefits [8] [10]. After the procedure, initial contrast-enhanced MRI or CT is generally used to assess the ablation zone, followed by regular long-term imaging to check for recurrences [8].

Discussion 

Histotripsy, even in its experimental phase, has surfaced as an alternative to other modalities such as thermal ablation, RFA, and MWA [10]. Because no treatment is providing the necessary local control, novel ways to treat liver metastasis and primary cholangiocarcinoma are always coming up [11-13]. MWA and SBRT use heat or radiation to cause coagulative necrosis, but histrotripsy is not the same. It is a non-thermal, non-ionizing, ultrasound-based technique that breaks down targeted tumours into acellular debris by expanding and collapsing microbubbles [10]. The mechanical selectivity allows for the preservation of collagen-rich structures, such as blood arteries and bile ducts, thereby mitigating the heat sink effect that limits traditional methods [14]. The Theresa and Hope4Liver first-in-human trial feasibility trial has definitively demonstrated initial clinical safety and efficacy in liver metastasis and primary HCC [8,9]. It represents a significant step in the translation of histotripsy from bench to bedside. Preclinical studies have further demonstrated its immunogenic potential, showing dendritic cell activation, increased CD8⁺ T-cell infiltration, and reduced metastatic burden in immunocompetent animal models [10,12,13,15]. These results imply that histotripsy may have an abscopal-like immunological response, making it a link between local and systemic cancer treatment [10].

Many cases of primary HCC and liver metastases continue to occur due to comorbidities, cirrhosis, and proximity to blood vessels, despite the availability of various liver-directed treatments. Histotripsy can greatly enhance local control and overall survival in this type of metastatic disease through its unique working method and immune-mediated response. It seems to be a non-thermal and non-invasive choice for definitive treatment, working with systemic treatment and as a bridge to a liver transplant. Because of this, it could also be used with immunotherapy and added to a multimodal liver treatment plan.

Early results have proven promising, even with these limitations. Histotripsy worked well and was quite safe, especially in places where the anatomy was difficult [11]. There were very few complications. The most prevalent ones were temporary increases in liver enzymes and mild discomfort [8]. There were no reports of bile duct strictures, significant bleeding, or systemic toxicity [9]. This outstanding safety record makes it especially appealing for people with cirrhosis or low liver reserves, for whom thermal or surgical treatments are dangerous. The non-thermal approach may also work well with immunotherapy or systemic treatments. This is because breaking up the tumour mechanically releases intact antigens that could make checkpoint inhibitors work better. But in the real world, how well it works will depend a lot on money and logistics. The histotripsy device costs between $300,000 and $500,000, and it needs anaesthesia, which makes it pricier at first. But its ability to cut down on hospital stays, avoid costs for items that need to be replaced, and lower the risk of complications may make the price worth it in busy centers. The FDA approved the method in 2023 for liver tumors, and it is now being used in some places in North America, Europe, and Asia. In nations with low or intermediate incomes, implementation will depend on partnerships between schools, shared infrastructure, and well-organized training programs.

Histotripsy is not likely to replace traditional ablation; instead, it will complement it. It is excellent for lesions close to blood vessels or biliary structures, tumours that come back or stay after RFA/MWA, and individuals who can't have invasive procedures. As data accumulates, it may develop into a bridging or combinatorial therapy alongside systemic or immunological medicines.

Future studies should look at long-term cancer outcomes, how well the results can be replicated at different institutions, cost-effectiveness evaluations, and better imaging biomarkers for therapy response. Developing simpler delivery systems, ones usable under light sedation and guided by a real-time targeting tool, could help histotripsy become more practical and accessible globally.

## Conclusions

Histotripsy represents a major advancement in the management of liver malignancies. By employing focused mechanical cavitation rather than thermal or ionizing energy, it achieves precise and complete tumor disintegration while preserving vital vascular and biliary structures. Early studies confirm its safety, minimal invasiveness, and reproducibility across centers, with promising immune-modulating effects that could complement systemic therapies. In the future, researchers need to concentrate on how histotripsy works in the long term and what really makes a difference in survival and quality of life for patients. Having definite standards of treatment will facilitate the use of this technology by clinicians with confidence. Meanwhile, enhancing local and systemic control of tumors by combining histotripsy with immunotherapy or drugs targeted at cancers may become possible. As the technology evolves into something simpler and more accessible, collaboration between hospitals and research teams will be the key to making histotripsy a cheap and viable option for the treatment of liver cancer sufferers across the globe.
